# Structural Cardiac Abnormalities, Ventricular Dysfunction Phenotypes, and Heart Failure Risk among Antiretroviral Therapy-treated People Living with HIV in South Africa

**DOI:** 10.64898/2026.06.04.26354960

**Published:** 2026-06-08

**Authors:** Zaayid Omar, Abdullahi M. Ahmed, Julian Wolfson, Zuofu Huang, Msimelelo Mgidlana, Ashley Black, Marya Abd El Hadi, Olukayode O. Aremu, Tess Peterson, Ntobeko A. B. Ntusi, Graeme Meintjes, Mpiko Ntsekhe, Jason V. Baker

**Affiliations:** 1 – Centre for Infectious Diseases Research in Africa, Institute of Infectious Disease and Molecular Medicine, University of Cape Town, South Africa; 2 – Division of Infectious Diseases, Hennepin Healthcare Research Institute, Minneapolis MN, USA; 3 - Division of Biostatistics & Health Data Science, School of Public Health, University of Minnesota, Minneapolis MN, USA; 4 – Division of Cardiology, Department of Medicine, University of Cape Town, Cape Town, South Africa; 5 - Midwestern University, Downers Grove IL, USA; 6 – Cape Heart Institute, University of Cape Town, South Africa; 7 – Division of Epidemiology and Community Health, School of Public Health, University of Minnesota, Minneapolis MN, USA; 8 – South African Medical Research Council, South Africa; 9 – Division of Infectious Diseases and HIV Medicine, Department of Medicine, University of Cape Town, Cape Town, South Africa; 10 – Blizard Institute, Faculty of Medicine and Dentistry, Queen Mary University of London, London, UK; 11 – South Africa Medical Research Council Extramural Unit for Intersection of Infectious and Non-Communicable Diseases, South Africa; 12 – Department of Medicine, University of Minnesota, Minneapolis MN, USA

## Abstract

**Background:**

The manifestations of cardiovascular disease (CVD) among people with HIV (PWH) differ by region globally. While HIV disease is associated with increased atherosclerotic CVD risk in the global North, non-ischemic heart failure (HF) is more common in sub-Saharan Africa, the global HIV epicenter. We estimated the effect of treated HIV on the frequency and phenotype of HF and its cardiac precursors in South Africa (SA).

**Methods:**

In an observational study, we recruited PWH on antiretroviral therapy (ART), age ≥40 years and people without HIV (PWoH) with similar distributions of age, sex, ethnicity, and hypertension, from a community clinic in Khayelitsha (Cape Town, SA). Procedures included a clinical assessment, echocardiography (Echo), and b-type natriuretic peptide (BNP) measure. Echo parameters defined structural abnormalities, left ventricle (LV) filling pressure, and LV systolic and diastolic dysfunction (DD). HF was defined by symptoms and/or BNP ≥35pg/mL and LV dysfunction, subcategorized as reduced, mildly reduced, or preserved ejection fraction (HFrEF, HFmrEF, and HFpEF). Comparisons by HIV status were adjusted for age, sex, hypertension, smoking, obesity, diabetes, elevated LDL-cholesterol, and hazardous alcohol use.

**Results:**

Between September 2022 and August 2025, we enrolled 1008 PWH and 500 controls [median (Q1-Q3) age 48 years (43–53), 77% female]. Among PWH and controls respectively, 37% and 39% had hypertension, 21% and 25% were current smokers, 40% and 45% were obese, and 9% and 17% had diabetes. LV systolic dysfunction (1%) and HFrEF (1%) were rare, and undiagnosed HFpEF (8%) was the predominant HF phenotype. Compared to controls, PWH had higher odds of elevated LV mass index (LVMI) (OR 2.1; 95%CI 1.5–3.0) and DD (OR 1.4; 95%CI 1.0–2.0). Risk for elevated LVMI and DD was greatest among women with HIV, who also had an increased risk for undiagnosed HFpEF (OR 1.9; 95%CI 1.2–3.2), compared to women without HIV; effects which were not seen among men (p=0.051 for HIV*Sex interaction).

**Conclusions:**

In a peri-urban SA community with a high burden of cardiometabolic risk factors, the frequency of abnormal structural and functional cardiac precursors of HFpEF was greater amongst ART-treated PWH. This was most pronounced amongst women with HIV, who also had increased risk of undiagnosed HFpEF.

## Introduction:

HIV remains a major contributor to morbidity and mortality in sub-Saharan Africa, where the majority of people with HIV (PWH) reside globally^1^. Widespread access to anti-retroviral therapy (ART) has led to prolonged survival and reduced risk for AIDS complications, but the relative proportion of deaths and disability attributable to age-related chronic conditions such as cardiovascular disease (CVD) has increased, and these conditions present at earlier ages.^2^ It has been estimated that ART-treated PWH have a twofold higher risk of developing both atherosclerotic CVD (ASCVD) events and heart failure (HF).^3^ However, epidemiologic data describing the changing spectrum of HIV-associated comorbid conditions largely arise from high income countries (HICs) in the global North, and the generalizability of these data to sub–Saharan Africa (SSA) is unclear.^4^

In SSA, data suggests that the spectrum and burden of CVD amongst both PWH and people without HIV (PWoH) is different from HICs, with non-ischemic CVD manifestations playing a more prominent role.^5–8^ Much of the published literature on HIV-related CVD from SSA predates current clinical practice with effective ART treatment, is based on descriptions of risk factor prevalence^9,10^, and/or relies on epidemiological models informed by data from HICs to estimate HIV-associated CVD risk in low-middle income countries.^3^ More recent data suggests that, when compared to HICs, SSA has much lower rates of ASCVD and its complications^5,11^ and higher rates of HF due to non-ischemic causes such as hypertension, primary valve pathology, and heart muscle disease.^12^ Importantly, when considering HIV-associated CVD between HICs and SSA, there are major differences in population characteristics, social determinants of health, and burden of non-traditional CVD risk factors such as tuberculosis.^13^

Here we report the primary findings from the Prevalence and Phenotype of HIV-HFpEF in South Africa (PHIZA) study. This study provides contemporary data within a front-line clinical setting in SSA on the contribution of HIV to HF risk, including the frequency of cardiometabolic risk factors influencing HF risk, the spectrum of structural and functional cardiac abnormalities, the frequency of undiagnosed clinical HF and the underlying phenotypes within a high HIV prevalence region in the global South.

## Methods:

### Study Design and Population

PHIZA was an observational study of PWH and PWoH designed to determine the prevalence of major heart muscle and cardiac chamber abnormalities, and HF phenotypes in a SSA community with a high prevalence of HIV and tuberculosis. Study participants were enrolled at the Site B Community Health Center clinic in Khayelitsha, a low socio-economic peri-urban town outside of Cape Town, SA. Eligibility criteria included PWH who were 40 years and older, on ART for at least one year without interruption, and virally suppressed (HIV viral load level <200 copies/mL) on their most recent clinical laboratory evaluation. Control participants were PWoH from the same community with recruitment targeting similar frequencies of age ≥50 years, female sex, and hypertension status, to prevent large differences between groups in these potential confounders. Key exclusions included women who were pregnant or lactating, active tuberculosis or bacterial infection, and chemotherapy treatment for cancer within the prior six months.

The study was approved by the health research ethics and human subject research committees at the University of Cape Town [HREC Reference 119/2022] and Hennepin Healthcare Research Institute, respectively. All participants underwent verbal and written informed consent procedures in isiXhosa, Afrikaans or English, as appropriate.

### Study Assessments

**Figure 1:**
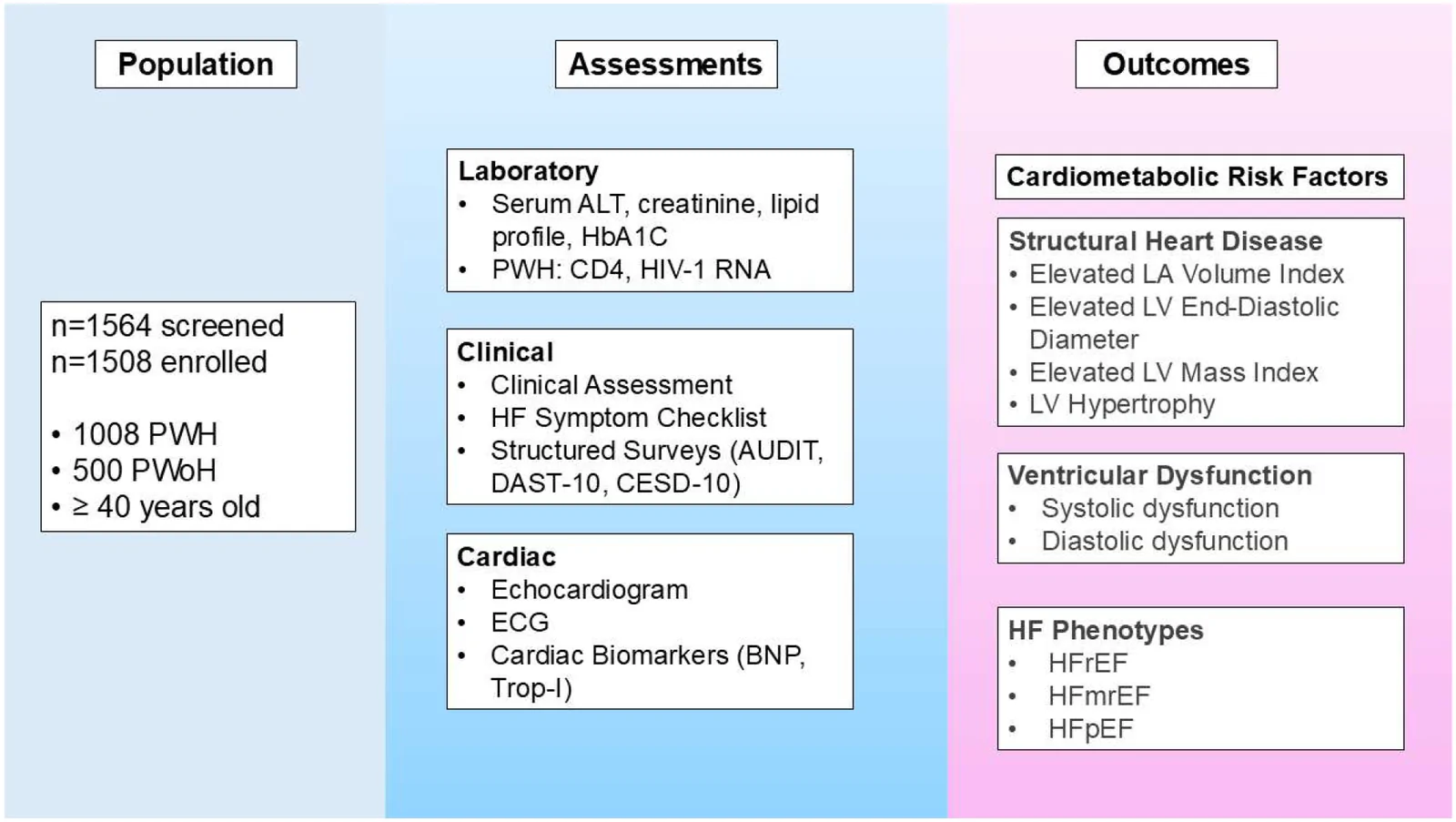
Study design schematic ALT=alanine aminotransferase; AUDIT=Alcohol Use Disorders Identification Test; BNP=b-type natriuretic peptide; CESD-10=Center for Epidemiologic Studies Depression Scale-10; DAST-10=Drug Abuse Screen Test-10; ECG=electrocardiogram; HbA1C=hemoglobin A1C; HFmrEF=heart failure with mildly reduced ejection fraction; HFpEF=heart failure with preserved ejection fraction; HFrEF=heart failure with reduced ejection fraction; LA=left atrium; LV=left ventricle; PWH=people with HIV; PWoH=people without HIV; Trop-T=Troponin-I

Participants underwent clinical, cardiac, and laboratory assessments as described in Figure 1. Symptoms suggestive of HF were solicited via a structured 5-question checklist that asked about recent shortness of breath, dyspnea on exertion, nocturnal dyspnea, fatigue, and lower limb swelling (see Supplemental Material for full list of Supplemental Methods). Additional cardiac assessments were conducted in the clinic setting and included a blood pressure, electrocardiogram, echocardiogram (ECHO), and point of care blood testing for B-type natriuretic peptide (BNP) and Troponin-I levels (Quidel Alere^™^ Triage^®^ MeterPro). ECHO imaging was performed by an experienced cardiac ultrasound technician and approximately one-third of images (n=425) were reviewed remotely by a cardiologist for quality assurance, including all participants with features of possible diastolic dysfunction and a random sample of patients without LV dysfunction.

Structured behavioral surveys were conducted including the Alcohol Use Disorders Identification Test (AUDIT), Drug Abuse Screening Test-10 (DAST-10), and Center for Epidemiological Studies Depression Scale-10 (CESD-10).

### Outcomes

Cardiometabolic risk factors of interest included hypertension (known diagnosis on medical chart review, use of antihypertensive therapy, or elevated blood pressure at recruitment [systolic BP>160 or diastolic BP > 100]), obesity (BMI ≥30 kg/m^2^), diabetes mellitus (known diagnosis, use of antidiabetic therapy, or serum hemoglobin A1C level ≥6.5% at recruitment), increased low density lipoprotein cholesterol (LDL-c) (LDL-c level ≥130 mg/dL [3.36 mmol/L] or use of lipid lowering therapy), chronic kidney disease (CKD stage IIIa or higher based on estimated glomerular filtration rate), and coronary artery disease (known diagnosis). Depressive symptoms were defined by a score of ≥10 on the CESD-10 scale, hazardous or harmful alcohol use defined by a score of ≥8 on the AUDIT scale, and moderate or greater illicit substance abuse by a score of ≥3 on the DAST-10 scale.

The American Heart Association (AHA) progressive HF stages were characterized, consisting of: Stage A, defined by the presence of cardiometabolic risk factors for HF without evidence of structural or functional cardiac abnormalities; Stage B, defined by ECHO evidence of either LV systolic or diastolic dysfunction; and Stage C (clinical HF), which included people with HF symptoms (from 5-question checklist) or BNP level of ≥35 pg/mL, and echo abnormalities from stage B. No participants in our sample were classified as advanced HF (Stage D) - these patients are immediately referred to a specialty cardiology unit and would not typically receive care at the recruitment site.

The heart disease outcomes of interest include structural cardiac abnormalities, ventricular dysfunction, and clinical HF definitions (detailed criteria in Table 1). Standardized published ECHO criteria were used to define the cardiac structural and functional abnormalities, and increased LV filling pressures.^14^

### Statistical Analysis

At an estimated prevalence of HF and ventricular dysfunction in the range of 5–30%, our sample size was calculated at 1000 PWH and 500 PWoH to estimate prevalence with a margin of error of < 4 percentage points and yield 80% power to detect a difference of 7 percentage points by HIV status.

Participant characteristics, ECHO parameters and additional outcomes were summarized by median (Q1-Q3) for continuous variables and proportion (%) for categorical variables. Outcomes were compared between groups using univariable and multivariable logistic regression models including the following hypothesized confounding factors: age, sex, hypertension, smoking, obesity, diabetes, elevated LDL-c, and harmful/hazardous alcohol use. Comparisons by HIV status were then evaluated separately for men and women. Models also considered multiplicative interaction terms for HIV-by-sex and HIV-by-age.

## Results

From September 2022 to July 2025, 1508 participants were enrolled (1008 PWH and 500 PWoH). Only participants with complete echo data were included for analysis. Participant characteristics are presented in Table 2 and HIV-specific factors in Table 3. The median age of all participants was 48 years, 77.3% were female, and the frequencies of hypertension (37.7%) and obesity (41.5%) were high, while frequencies of previously diagnosed coronary artery disease (0.4%) and chronic kidney disease (0.3%) were low. Among PWH, the majority (73.7%) had been living with HIV for over 10 years, 96.4% had a suppressed (<200 copies/mL) HIV RNA level at enrollment, and 37.9% had a nadir CD4 count <200 cells/μL. The median current CD4 count was 588 cells/μL, and the frequency of previous TB disease was 3-fold higher among PWH vs. PWoH (48.9% vs 16.0%, respectively).

Overall, 65% of participants had at least one cardiometabolic risk factor. The frequency of hypertension was similar (37.2% in PWH vs 39% in PWoH) in both groups by intention, but PWoH more commonly had obesity (39.7% vs 45.2%), diabetes (8.7% vs 17.2%) or elevated LDL-c levels (10.4% vs 17.6%).

Classification of progressive AHA HF stages is shown in Table 4. Both groups had similar frequencies of participants without risk factors or evidence of structural heart disease (no AHA stage), though AHA Stage A was more common among PWoH (27.1% vs 36.8%; p<0.001). However, compared to PWoH, the proportion of AHA Stage B (structural or functional heart disease without symptoms) was significantly higher among PWH (38.9% vs. 32.0%; p=0.011), while AHA Stage C (clinical HF) (10.5% vs 9.0%; p=0.405) was not significantly different.

**Figure 2:**
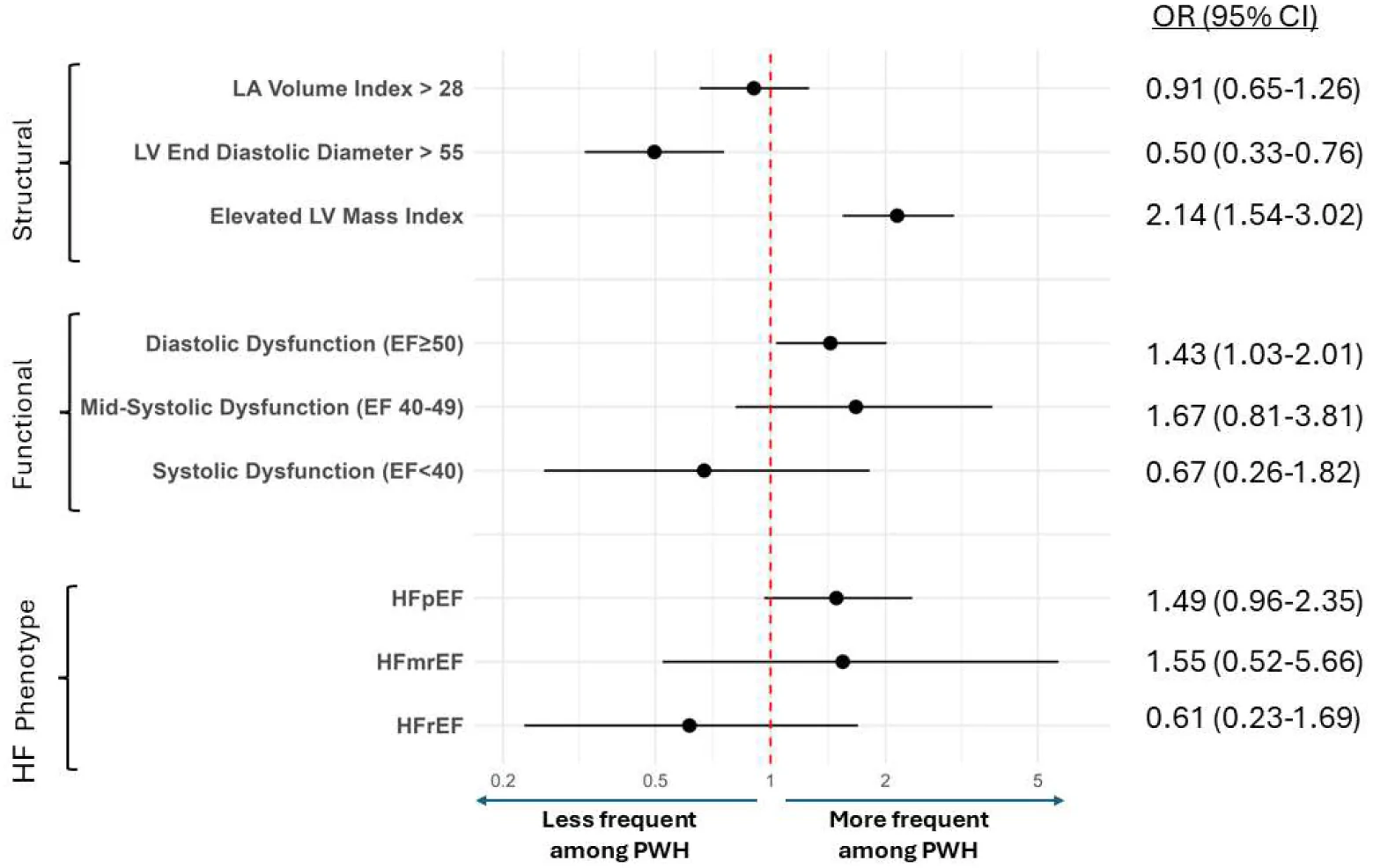
OR (95% CI) for myocardial abnormalities among people with vs without HIV OR (95% CI) plotted for each cardiac variable comparing people with vs. without HIV (PWH, PWoH). OR adjusted for age, sex, diabetes, hypertension, elevated LDL-cholesterol (LDL-c ≥ 3.362 mmol/L), smoking and hazardous alcohol use. HF=heart failure; HFmrEF=Heart Failure with Mildly Reduced Ejection Fraction; HFpEF=Heart Failure with Preserved Ejection Fraction; HFrEF=Heart Failure with Reduced Ejection Fraction; LA=left atrium; LV=left ventricle.

Frequencies of cardiac structural abnormalities, LV dysfunction, and HF syndromes are reported in **Supplemental Table 1**, and the risk of these outcomes by HIV status is presented in Figure 2. When compared to PWoH, PWH were significantly less likely to have elevated LVEDD (5.1% in PWH vs 10.0% in PWoH; OR 0.50; 95% CI 0.33–0.76; p<0.01), and more likely to have increased LVMI (19.9% in PWH vs 11.6%; OR 2.14; 95% CI 1.54–3.02 p<0.001) and LV wall thickness (20.6% vs 7.6%; OR 3.76; 95% CI 2.60–5.58; p<0.001). Systolic dysfunction did not differ, but diastolic dysfunction was more frequent among PWH compared to PWoH (15.8% vs. 12.8%; OR 1.43; 95% CI 1.03–2.01; p=0.033) (**Supplemental Table 1**).

Overall, HFpEF was the predominant HF phenotype (7.8%) while HFrEF was rare (1.1%). Compared to PWoH, HFpEF was more frequent among PWH (8.4% vs 6.6%) though this difference did not reach statistical significance in unadjusted (OR 1.30; 95% CI 0.87–2.0; P=0.21) or adjusted (OR 1.49; 95% CI 0.96–2.35; p=0.08) models. Other factors associated with HFpEF included older age, female sex, hypertension, and current or past smoking (Table 5). When considering HIV-specific factors among PWH, risk for HFpEF was associated with having a current CD4 count <200 cells/μL (OR 4.49; 95% CI 1.68–10.8; p<0.01; **Supplemental Table 2**). Exposure to protease inhibitor–containing ART regimens also had a large but non–significant point estimate for an association with HFpEF (OR 1.86; 95% CI 0.88–3.62; p=0.082). There was no association with duration of HIV infection, duration of ART use, or exposure to integrase strand transfer inhibitor–containing ART regimens. No TB–specific factors were significantly associated with HFpEF (**Supplemental Table 3**).

**Figure 3:**
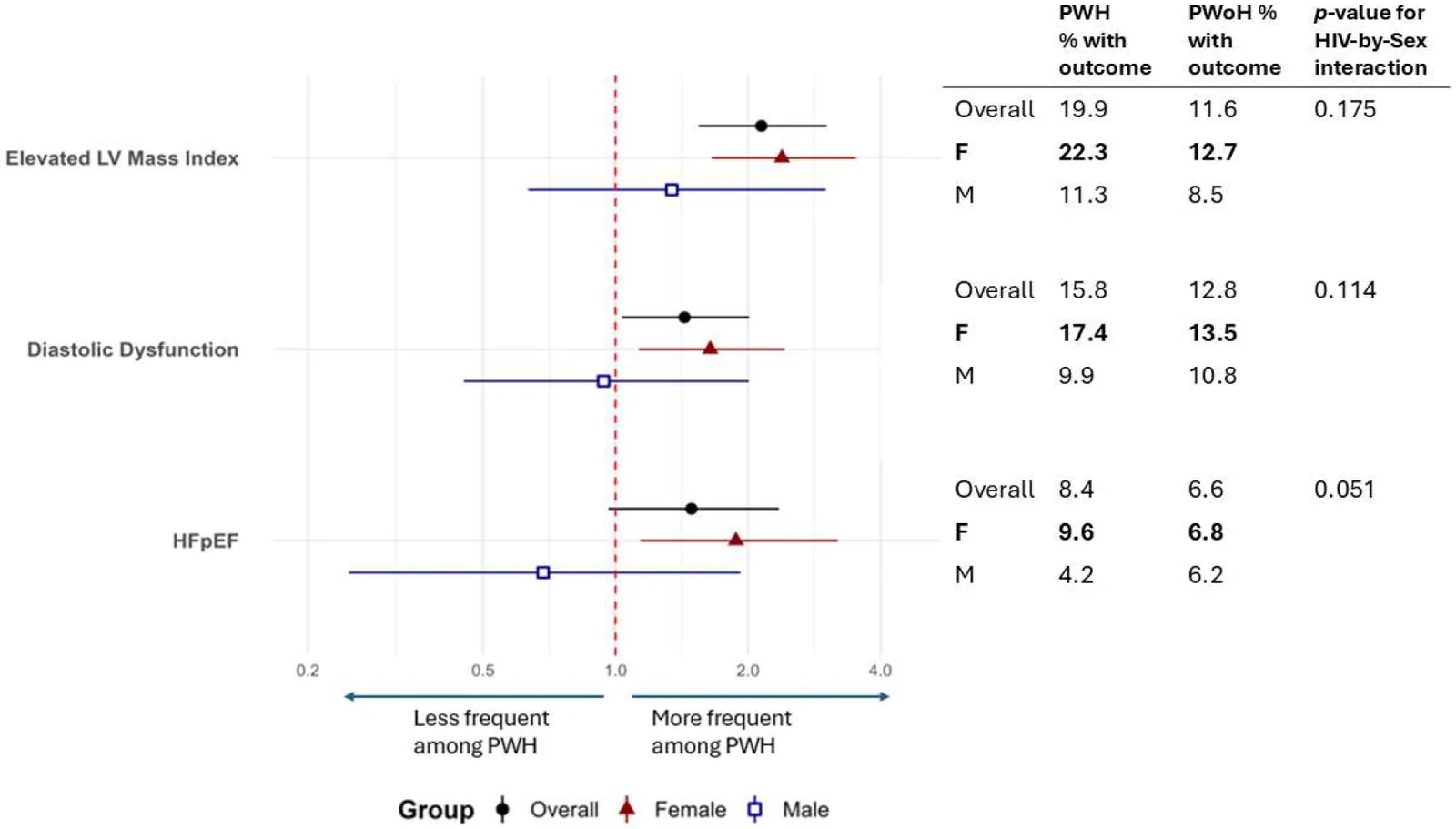
Risk for myocardial abnormalities by HIV status and sex OR (95% CI) plotted for each cardiac variable comparing people with vs. without HIV (PWH, PWoH), separated by sex. OR adjusted for age, diabetes, hypertension, elevated LDL-cholesterol (LDL-c ≥3.362 mmol/L), smoking and hazardous alcohol use.

The potential influence of sex on HFpEF associations with HIV status was further examined, given substantial differences in frequencies of hypertension (27.1% vs 40.9%), obesity (6.7% vs 51.8%) and diabetes (5.0% vs 13.5%) between males and females, respectively (**Supplemental Tables 4 and 5**). When comparisons were restricted to females, the magnitude of increased risk among PWH was greater for LVMI (OR 2.39; 95% CI 1.65–3.51; p<0.001), LV wall thickness (OR 4.47; 95% CI 2.84–7.33; p<0.001), and diastolic dysfunction (OR 1.64; 95% CI 1.13–2.42; p=0.011), when compared to estimates among men or among all participants (Figure 3 and **Supplemental Tables 6 and 7**). Risk for HFpEF was now significantly higher for women with HIV compared to women without HIV (OR 1.88; 95% CI 1.14–3.20; p=0.016). Further, there was suggestion of heterogeneity in the effect of HIV on risk for HFpEF between females and males (p–value for the HIV–by–sex interaction = 0.051). HIV–by–age (p=0.953) and HIV–by–menopausal status (p=0.973) interaction terms were not significant.

## Discussion

We described the frequency of structural and functional cardiac abnormalities, and undiagnosed HF phenotypes, among a population of ART-treated PWH and PWoH in a front-line clinical setting within Khayelitsha, South Africa (SA). There are four primary findings: (1) Traditional cardiometabolic risk factors (e.g., hypertension and obesity) and subclinical cardiac abnormalities (e.g., LV wall thickness) characterized by ECHO were very common among both persons with and without HIV; (2) The overall frequency of undiagnosed AHA HF stages B and C was unexpectedly high, and amongst those with AHA HF stage C, HFpEF was the predominant HF phenotype while HFrEF was rare; (3) Compared to PWoH, PWH had a higher frequency of ECHO-determined LV wall thickness and increased LV mass, as well as diastolic dysfunction, both of which often precede and contribute to clinical HFpEF risk; and (4) in this community, the overall frequencies of structural and functional cardiac abnormalities and risk for undiagnosed HFpEF were significantly higher among women with HIV versus women without HIV, independent of cardiometabolic risk factors.

While there is wide acceptance that CVD risk is increased among PWH, most of the evidence to date focuses on coronary atherosclerosis and its associated ischemia-induced clinical manifestations.^15^ Accelerated atherogenesis among PWH is multifactorial, but is due, in part, to high prevalence of cardiometabolic risk factors, adverse effects and toxicities of antiretroviral drugs, and persistent systemic inflammation and immune activation despite plasma viral suppression with ART.^16–19^ However, the degree to which these global North derived data are generalizable to regions such as SSA, where epidemiologic data for CVD in the general population suggest a predominance of non-ischemic heart disease that is less influenced by coronary atherogenesis per se, is unclear,.^6–8,20^

To our knowledge, this is the first SA based study to describe the prevalence of HF and HF phenotypes among an otherwise healthy community population including PWH receiving effective ART treatment. The high prevalence of traditional cardiometabolic risk factors among PWH in this study was consistent with data from other SSA cohorts (comparisons presented in **Supplemental Table 8**). However, compared to studies conducted in the global North (**Supplemental Table 9**), PWH in PHIZA had less frequent elevations in non-HDL cholesterol and more frequent obesity and hypertension. Both obesity and hypertension are not only risk factors for heart failure but may be causally related,^21–23^ potentially contributing to the high prevalence of undiagnosed HFpEF overall in PHIZA.

Data from the global North have also shown that HIV is associated with increased risk for both HFrEF and HFpEF phenotypes.^24^ HIV-associated HF in the pre-ART era, with advanced untreated HIV/AIDS, was typically characterized by myocarditis and myonecrosis leading to dilated cardiomyopathy with a reduced LV systolic function.^25^ With the widespread use of ART and associated viral suppression and immune recovery, HIV-associated dilated cardiomyopathy is less common in contemporary settings. Recent ECHO data among ART-treated PWH with high CD4 counts suggest a higher burden of diastolic dysfunction.^26,27^ In PHIZA, HFpEF was the predominant HF phenotype regardless of HIV status. Further, when compared to controls, PWH had a 171% higher risk of having increased LV wall thickness, 71% higher risk of having increased LV mass, and 23% higher risk for diastolic dysfunction, all precursors to HFpEF.

Comparisons of HF phenotypes with other data from SSA are difficult due to the lack of population-based studies with detailed echocardiographic assessments. Prior epidemiologic data from SSA support a similar predominance of non-ischemic CVD manifestations including HF phenotypes, but these data are either outdated (e.g., from the pre-ART era among those with advanced AIDS) or have design limitations (such as small sample sizes, mixed reporting of ART use, or reporting surrogate CVD markers without clinical CVD phenotypes).^10,12,28^ For example, the Heart of Soweto^20^ (which assessed 4810 patients of which 9.7% were PWH) was designed to determine the etiology of CVD in a peri-urban Johannesburg (SA) community with a similar demographic and socioeconomic background to Khayelitsha and similar burden of HIV.^29^ Non-ischemic HF was the most common CVD manifestation (44%), while ASCVD complications, such as ischemic heart disease (2.7%) and cerebrovascular disease (3.5%) were infrequent. Further analyses from the Heart of Soweto study, limited to only participants with HIV, found ASCVD-related admissions in only 2.4%.^28^ Consistent with this, emerging CVD epidemiology from the general population in SSA report the most common causes of HF as hypertensive heart disease, cardiomyopathy, and rheumatic heart disease^6^, with limited contribution from coronary artery disease.^5,6,8^ These findings highlight the importance of contemporary studies from the global South like PHIZA, and the potential hazard of relying on information derived from populations defined by a different spectrum of risk factors.

Possible mechanisms contributing to risk for HFpEF among PWH receiving effective ART include HIV-related systemic inflammation and structural changes characterized by excess myocardial fibrosis and steatosis.^15^ Evidence of HIV-associated myocardial fibrosis and steatosis has now been reported in numerous studies utilizing cardiac magnetic resonance imaging, as well as histopathology from a sentinel autopsy study.^15,30–33^ Among PWH, myocardial fibrosis has also been associated with circulating proinflammatory monocytes and a plasma proteomic signature enriched for T cell activation and TNF signaling, suggesting a possible immune mechanism for cardiac inflammation.^32,34,35^ Finally, multiple studies from the general population have reported associations for diastolic dysfunction and risk for HFpEF with systemic inflammation, endothelial dysfunction, and myocardial steatosis, all of which are accentuated among PWH.^17,21,36–38^

Our findings build on existing literature by identifying that women with HIV may have a higher frequency of undiagnosed HFpEF compared to women without HIV, whereas this effect may not be present among men (Figure 3; **Supplemental Tables 6 and 7**). In addition to having higher frequencies of hypertension and obesity, both key risk factors for HFpEF, the association between HIV status and increased LV mass, LV hypertrophy, and diastolic dysfunction was more prominent among women. (Figure 3; **Supplemental Tables 4 and 5**). This is consistent with a recent analysis of the global REPRIEVE trial which reported that incident HF was higher in women and those with hypertension and obesity.^39^ Furthermore, outside of HICs, incident HF was reported more frequently in SSA than other regions in REPRIEVE.^39^ Among a study of out of hospital cardiac arrest in the U.S., PWH were at increased risk for sudden cardiac death when compared to persons without HIV in the same community, and this effect was greater among women than men.^33^ Finally, the observation that the relative increase in CVD risk from HIV is greater among women, compared to men, has also been repeatedly shown for ASCVD events.^40,41^ Some have posited that sex differences in HIV-related CVD may be due to underlying differences in immune activation, which could have implications for both ischemic and non-ischemic CVD manifestations.^42^ Future research is needed to better characterize the potential interaction between HIV disease, obesity, and female sex, as well as the associated mechanisms contributing to LV wall thickness, diastolic dysfunction and clinical risk from HFpEF.

The clinical syndrome of HFpEF has been characterized as the end result of cardiac insults and stress from numerous heterogeneous comorbidities.^21^ Recently, a novel unifying biologic framework has been proposed that describes the pathogenesis of HFpEF arising from dysfunctional adipose tissue rather than as a primary disorder of cardiomyocytes.^22^ This framework has been referred to as the “adipokine hypothesis”, which states that an expansion of altered visceral adipose tissue leads to an increased and altered profile of adipokine release into the circulating blood volume, which over time contributes to systemic inflammation, plasma volume expansion and cardiac hypertrophy and fibrosis.^22^ In this context, women have increased adipokines and related inflammatory markers when compared to men.^43^ Further, women are at a two-fold increased risk for HFpEF when compared to men, and obesity poses greater risk for HFpEF among women when compared to men.^44^ Applying the 'adipokine hypothesis’ to PWH in the current era, it is notable that chronic inflammation and adiposity may both be more prominent among women when compared to men with HIV.^42,45^ Specific to ART treatment, weight gain has been associated with integrase strand transfer inhibitor-based regimens, with the greatest weight gain reported among black women.^45^ In summary, our findings suggest that ART-treated HIV disease may amplify key components of the ‘adipokine hypothesis’, namely inflammation and visceral adiposity, thereby accentuating the pathogenesis of HFpEF particularly among women with HIV.

This study had several important limitations. The cross-sectional design inherently limits causal inference and may also include unmeasured confounding. The clinical and laboratory assessments were limited within the frontline clinic research setting, which prevented exploration of immunologic and inflammatory pathways that may be contributing to the cardiac outcomes described. Finally, the study population age was primarily between 40 to 60 years old, but this reflects an age range at higher risk for CVD where prevention strategies are also still useful. The study population was predominantly women, reflective of the epidemiology within SA, with approximately two thirds of adult PWH in SA being women^29^; nonetheless, this limited our ability to make comparisons restricted to men only. Additionally, comparisons between men and women were potentially confounded by meaningful differences in the profile of cardiometabolic risk factors.

## Conclusion

In a peri-urban South African (SA) community with low socioeconomic status and a high prevalence of HIV, TB and cardiometabolic risk factors, undiagnosed HFpEF was common and was the predominant HF phenotype. HIV was associated with increased risk for subclinical structural and functional cardiac abnormalities including greater LV mass, LV wall thickness, and diastolic dysfunction, which all contribute to HFpEF risk. These findings were more prominent among women with HIV when compared to women without HIV. In this context, PWH in SA, particularly women, have an increased risk for HF that is characterized by thick, stiff myocardium, independent of cardiometabolic risk factors. This large community-based study adds to increasing evidence suggesting that in SSA, where the prevalence of HIV is high and the burden of ASCVD is relatively low,^5^ the absolute excess CVD risk due to HIV manifests most frequently as non-ischemic HF and HFpEF. Further research should focus on the potential interaction and interconnectedness of HIV disease, female sex and obesity along with their influence on HFpEF pathogenesis.

## Supplementary Material

- Supplemental Material including Supplemental Methods
- Tables S1–S11
- Figure S1
- References 46–50

## Figures and Tables

**Table 1:** Outcome Definitions for Structural Cardiac Abnormalities, Ventricular Dysfunction, and Heart Failure Phenotypes

Outcome	Definition
**Structural Cardiac Abnormalities**	Echocardiographic Criteria
Left Atrial Enlargement	LAVI >28mL/m2
Left Ventricular Dilation	LVEDD >55mm
Elevated Left Ventricular Mass (LVM)	LVMI >115g/m2 (males) or >95g/m2(females)
Left Ventricular Hypertrophy (LV wall thickness)	LVPWd or IVSd >11mm
**Left Ventricular Dysfunction**	Echocardiographic Criteria
Diastolic Dysfunction	LV EF ≥50% and ≥1 criteria from both A & B: Increased LV filling pressure E/e’ >9 Septal e’ <8 TR velocity >2.8 m/s AND LAVI >28 mL/m2 Structural cardiac changes LAVI >28mL/m2 LVMI >115g/m2 (Males), or >95g/m2 (Females)
Systolic Dysfunction (mid-range EF)	LV EF=40–49%
Systolic Dysfunction (reduced EF)	LV EF<40%
**Heart Failure (HF) Phenotypes**	Clinical and Echocardiographic Criteria
HFpEF	HF symptoms or BNP>35pg/mL, and diastolic dysfunction, and the absence of: *significant valvular disease, pericardial disease, congenital heart disease, isolated RV failure, PHTN, and COPD*.
HFmrEF	HF symptoms or BNP>35pg/mL, and LV EF=40–49% the absence of: *significant valvular disease, pericardial disease, congenital heart disease*
HFrEF	HF symptoms or BNP>35pg/mL, and LV EF<40% the absence of: *significant valvular disease, pericardial disease, congenital heart disease*

BNP=B-type Natriuretic Peptide; COPD=chronic obstructive pulmonary disease; E=early mitral inflow velocity; e’= early mitral annular diastolic velocity; EF=ejection fraction; HFmrEF=Heart Failure with Mildly Reduced Ejection Fraction; HF=heart failure; HFpEF=Heart Failure with Preserved Ejection Fraction; HFrEF=Heart Failure with Reduced Ejection Fraction; IVSd=end-diastolic interventricular septum; LAVI=left atrial volume index; LV=left ventricle; LVEDD=LV end-diastolic diameter; LVMI=LV mass index; LVPW=end-diastolic LV posterior wall; PHTN=primary pulmonary hypertension; RV=right ventricle; TR=tricuspid regurgitation

**Table 2:** Participant characteristics

	Median (Q1-Q3) or n (%)		
	Overall *n= 1508*	PWH *n = 1008*	PWoH *n=500*
Demographics			
Age, years	48 (43–53)	47 (43–52)	48 (43–56)
Sex at birth, female	1165 (77.3%)	795 (78.9%)	370 (74.0%)
Post-menopausal (females)	557 (47.8%)	368 (46.3%)	189 (51.1)
Cardiometabolic Characteristics			
Current smoker	333 (22.1%)	208 (20.6%)	125 (25%)
Body mass index, kg/m^2^	28.4 (23.2–33.6)	28.3 (23.6–33.1)	28.6 (22.5–34.9)
Waist-hip circumference ratio*	0.9 (0.84–0.95)	0.91 (0.85–0.96)	0.89 (0.84–0.94)
Obesity (BMI > 30 kg/m^2^), Overall	626 (41.5%)	400 (39.7%)	226 (45.2%)
Hypertension	569 (37.7%)	374 (37.1%)	195 (39.0%)
Diabetes mellitus	174 (11.5%)	88 (8.7%)	86 (17.2%)
Chronic kidney disease (stage ≥3)	4 (0.3%)	4 (0.4%)	0 (0%)
Coronary artery disease	6 (0.4%)	3 (0.3%)	3 (0.6%)
Cerebrovascular disease	31 (2.1%)	26 (2.6%)	5 (1%)
Total cholesterol (mmol/L†)	4.30 (3.74 – 4.94)	4.19 (3.62 – 4.85)	4.60 (4.0 – 5.16)
HDL-cholesterol (mmol/L†)	1.31 (1.09 – 1.62)	1.26 (1.07 – 1.51)	1.40 (1.17 – 1.76)
LDL-cholesterol (mmol/L†)	2.37 (1.85 – 2.93)	2.29 (1.80 – 2.86)	2.54 (1.95 – 3.08)
Elevated LDL-c (≥3.4 mmol/L†)	193 (12.9%)	105 (10.5%)	88 (17.6%)
TB History			
Prior clinical TB disease	573 (38.0%)	493 (48.9%)	80 (16.0%)
≥2 TB disease episodes	154 (10.2%)	139 (13.8%)	15 (3%)
Duration since 1st TB disease, yrs	14 (10–19)	14 (10–18)	15 (6–27)
Site of prior TB disease			
Pulmonary	477 (83.2%)	402 (81.5%)	75 (93.8%)
Extra-pulmonary	109 (16.8%)	103 (20.9%)	6 (7.5%)
Cardiac biomarkers			
BNP level >35 pg/mL	283 (18.8%)	184 (18.3%)	99 (19.8%)
BNP level >100 pg/mL	47 (3.1%)	32 (3.2%)	15 (3%)
Trop-I level detectable (>0.01 ng/mL)	47 (3.2%)	24 (2.4%)	23 (4.7%)
Substance use and mental health			
Hazardous or harmful alcohol use (AUDIT score ≥8)	426 (28.2%)	278 (27.6%)	148 (29.6%)
Moderate or greater problems from drug use (DAST-10 score ≥3)	22 (1.5)	16 (1.6)	6 (1.2)
Depressive symptoms (CESD-10 score ≥10)	186 (12.3)	98 (9.7)	88 (17.6)

* Available among 815 participants (n=404 PWH and n=411 PWoH);

† conversion factor to mg/dL = 38.67; AUDIT = Alcohol Use Disorders Identification Test; BMI = body mass index; BNP = b-type natriuretic peptide; CESD-10 = Center for Epidemiologic Studies Depression Scale-10; DAST-10 = Drug Abuse Screen Test-10; HDL = high-density lipoprotein; LDL = low-density lipoprotein; PWH = people with HIV; PWoH = people without HIV; TB = tuberculosis; Trop-T = Troponin-I

**Table 3:** HIV-specific participant characteristics

	Median (Q1-Q3) or n (%)
	PWH *n=1008*
HIV diagnosis duration, years	
<5 years	74 (7.3%)
5–10 years	191 (19.0%)
>10 years	742 (73.7%)
HIV diagnosis duration, years	14.3 (10–18)
Duration since ART initiation, years	12.0 (8–15)
HIV-1 RNA level <200 copies/mL	972 (96.4%)
HIV-1 RNA level <200 copies/m L for ≥3 years	680 (67.5%)
CD4 count	587.5 (425–746)
Nadir CD4 count <200 cells/μL	382 (37.9%)
Current ART use	
dolutegravir	959 (95.1%)
tenofovir disoproxil fumarate	939 (93.2%)
lamivudine	937 (96.5%)
abacavir	41 (4.1%)
Prior ART exposure	
efavirenz	886 (89.9%)
emtricitabine	865 (85.8%)
stavudine	205 (20.3%)
zidovudine	168 (16.7%)
ritonavir-boosted lopinavir	81 (8%)
abacavir	13 (1.3%)

ART = antiretroviral therapy; PWH = people with HIV; RNA=ribonucleic acid

**Table 4:** Classification across Mutually Exclusive AHA Heart Failure Stages

AHA Heart Failure Stage	*n* per stage	PWH *n=1008*	PWoH *n=500*	*p*-value
No stage	348	237 (23.5%)	111 (22.2%)	0.614
AHA Stage A (Risk factors* for heart failure with no evidence of disease)	457	273 (27.1%)	184 (36.8%)	<0.001
AHA Stage B (Echo abnormalities† but asymptomatic and normal BNP)	552	392 (38.9%)	160 (32%)	0.011
AHA Stace C (LV dysfunction with HF symptoms or BNP≥35pg/mL)	151	106 (10.5%)	45 (9%)	0.405

Stages are mutually exclusive.

* Risk factors included diabetes, hypertension, elevated LDL-cholesterol (LDL-c≥ 3.362 mmol/L), obesity, chronic kidney disease stages 3–5, and coronary artery disease.

† Echo abnormalities assessed include LA volume index, LV mass index, LV hypertrophy, LV end-diastolic diameter, systolic and diastolic dysfunction, and significant valvular disease. Stages are mutually exclusive. No participant was classified into Stage D. PWH=people with HIV; PWoH=people without HIV; AHA=American heart Association; BNP=B-type natriuretic peptide; LV=left ventricle

**Table 5:** Odds Ratios for HFpEF Risk in Multivariable Adjusted Model

	Odds Ratio (95% CI)	p value
HIV	1.49 (0.96–2.35)	0.080
Age per 10-year increase	1.82 (1.39–2.39)	<0.001
Female sex	2.34 (1.22–4.64)	0.012
Obesity	1.53 (0.99–2.36)	0.054
Hypertension	1.71 (1.12–2.61)	0.012
LDL-c ≥ 3.362 mmol/L	0.87 (0.46–1.53)	0.651
Diabetes	0.73 (0.39–1.31)	0.319
Smoking	1.75 (1.0–2.99)	0.043
Hazardous alcohol use	1.12 (0.66–1.86)	0.667

HFpEF risk adjusted for age, sex, obesity, diabetes, hypertension, elevated LDL-cholesterol (LDL-c≥ 3.362 mmol/L), smoking, and hazardous or harmful alcohol use

## Clinical Perspective

1. What is new?
  - In this community with a high prevalence of HIV, tuberculosis, and cardiometabolic risk factors, heart failure (HF) frequency (10%) was high and dominated by preserved ejection fraction (HFpEF) (8%).
  - Echocardiographic defined structural and functional precursors of HFpEF were comparatively more frequent in people with HIV,
  - Women with HIV were at highest risk of HFpEF.
2. What are the clinical implications?
  - There is a need for resource efficient tools for screening and early detection of HFpEF in primary care clinics serving similar communities.
  - The relationship between HIV, sex, and adiposity in HFpEF pathogenesis needs further study.

## Non-standard Abbreviations and Acronyms

AHA: American heart Association
ART: Antiretroviral therapy
ASCVD: Atherosclerotic cardiovascular disease
AUDIT: Alcohol Use Disorders Identification Test
BNP: B-type natriuretic peptide
CESD: Center for Epidemiological Studies Depression Scale-10
CVD: Cardiovascular disease
DAST-10: Drug Abuse Screening Test-10
DD: Diastolic dysfunction
ECHO: Echocardiogram
EF: Ejection fraction
HF: Heart failure
HFmrEF: Heart failure with mildly reduced ejection fraction
HFpEF: Heart failure with preserved ejection fraction
HFrEF: Heart failure with reduced ejection fraction
HIC: High income countries
LDL: Low density lipoprotein
LV: Left ventricle
LVMI: Left ventricle mass index
PWH: People with HIV
PWoH: People without HIV
SA: South Africa
SSA: sub-Saharan Africa
